# Supplementary material for: ‘Unmasking Pain’ through creativity: A phenomenological study of person-centred pain exploration for people living with chronic pain
Source: PLoS One. 2024 Oct 30;19(10):e0312014. doi: 10.1371/journal.pone.0312014 (PMC11524519; doi:10.1371/journal.pone.0312014)
Supplement: S1 File — (DOCX) [file pone.0312014.s001.docx]

# Interview guide – Unmasking Pain

**Introduction**

- Introduce the researcher
- Introduce the study topic
- Check consent and if participants are happy to continue
- Explain the aims and objectives of the study
- Explain confidentiality and anonymity
- Explain recording (audio-recorded) and length (1-1.5 hours), nature of discussion, outputs and data storage
- Check if participants have any questions

**General ‘rules’**

- Questions should be broad and open ended
- Questions be asked in the vocabulary and language of the individual being interviewed
- Don’t assume to understand what participants describe. Use clarifying questions

**Guiding questions** (choose as appropriate for the participant):

- **Explore the context of becoming involved in UP** e.g. Can you describe to me how you came to be involved in Unmasking Pain?
- **Explore the context of previous experience of pain** e.g. Can you describe to me your previous experiences of [living with or working/supporting or researching] pain?
  - Ask additional context questions in response to what the participants describe e.g. tell me more about [injury OR illness OR physiotherapy OR pain medication OR family OR work OR hobbies]
- **Explore the context of previous role in the Arts/Health/Pain** e.g. Can you describe to me your previous experiences of […]?
- **Descriptive question(s)** – e.g. can you describe a typical day at an Unmasking Pain workshop?
- **Descriptive question(s)** – e.g. can describe what happened when you attended the Unmasking Pain workshop?
  - Ask additional context questions in response to what the participants describe e.g. tell me more about […]
- **Structural question(s)** – You mentioned that the […] activity was […]. Can you describe to me what you mean by […]?
- **Clarifying question(s)** e.g. clarify to check understanding and avoid assumptions

**Wind down and summary**

- Leave space for questions and suggestions
- Are there any other points that participants would like to raise?
- Reiterate confidentiality/anonymity

# COREQ Checklist

| **No** | **Item** | | **Guide questions/description** | **Page reported** | |
| --- | --- | --- | --- | --- | --- |
| **Domain 1: Research team and reflexivity** | | | | |  |
| Personal Characteristics | | | | |  |
| 1. | Interviewer/facilitator | | Which author/s conducted the interview or focus group? | Page 6 (line 90) | |
| 2. | Credentials | | What were the researcher's credentials? *E.g. PhD, MD* | Title page | |
| 3. | Occupation | | What was their occupation at the time of the study? | Title page | |
| 4. | Gender | | Was the researcher male or female? | Not stated | |
| 5. | Experience and training | | What experience or training did the researcher have? | Included in sub section Strengths and weaknesses of the study, page 20 | |
|  | |  |  |  |  |
| 6. | Relationship established | | Was a relationship established prior to study commencement? | Included in section Materials and methods, page 6 | |
| 7. | Participant knowledge of the interviewer | | What did the participants know about the researcher? e*.g. personal goals, reasons for doing the research* | Included in section Materials and methods, page 6 | |
| 8. | Interviewer characteristics | | What characteristics were reported about the interviewer/facilitator? e.g. *Bias, assumptions, reasons and interests in the research topic* | Included in the sub-section Strengths and weaknesses of the study, page 20 | |
|  | |  |  |  |  |
|  | |  |  |  |  |
| 9. | Methodological orientation and Theory | | What methodological orientation was stated to underpin the study? *e.g. grounded theory, discourse analysis, ethnography, phenomenology, content analysis* | Included in section Materials and methods, Page 5 | |
|  | |  |  |  |  |
| 10. | Sampling | | How were participants selected? *e.g. purposive, convenience, consecutive, snowball* | Included in section Materials and methods, page 6 | |
| 11. | Method of approach | | How were participants approached? e*.g. face-to-face, telephone, mail, email* | Included in section Materials and methods, page 6 | |
| 12. | Sample size | | How many participants were in the study? | Included in section Results, page 8 | |
| 13. | Non-participation | | How many people refused to participate or dropped out? Reasons? | Included in section Results, page 8 | |
|  | |  |  |  |  |
| 14. | Setting of data collection | | Where was the data collected? e*.g. home, clinic, workplace* | Included in section Materials and methods, page 6 | |
| 15. | Presence of non-participants | | Was anyone else present besides the participants and researchers? | Included in section Materials and methods, page 6 | |
| 16. | Description of sample | | What are the important characteristics of the sample? *e.g. demographic data, date* | Included in section Results, page 8 | |
|  | |  |  |  |  |
| 17. | Interview guide | | Were questions, prompts, guides provided by the authors? Was it pilot tested? | Supplementary file | |
| 18. | Repeat interviews | | Were repeat interviews carried out? If yes, how many? | Included in section Results, page 8 | |
| 19. | Audio/visual recording | | Did the research use audio or visual recording to collect the data? | Included in section Materials and methods, page 6 | |
| 20. | Field notes | | Were field notes made during and/or after the interview or focus group? | Included in section Materials and methods, page 6 | |
| 21. | Duration | | What was the duration of the interviews or focus group? | Included in section Results, page 8 | |
| 22. | Data saturation | | Was data saturation discussed? | Included in section Results, page 8 | |
| 23. | Transcripts returned | | Were transcripts returned to participants for comment and/or correction? | Included in section Materials and methods, page 6 | |
|  | |  |  |  |  |
|  | |  |  |  |  |
| 24. | Number of data coders | | How many data coders coded the data? | Included in section Materials and methods, page 7 | |
| 25. | Description of the coding tree | | Did authors provide a description of the coding tree? | Included in section Results, page 10 | |
| 26. | Derivation of themes | | Were themes identified in advance or derived from the data? | Included in section Materials and methods, page 7 | |
| 27. | Software | | What software, if applicable, was used to manage the data? | Included in section Materials and methods, page 7 | |
| 28. | Participant checking | | Did participants provide feedback on the findings? | Included in section Materials and methods, page 6 | |
|  | |  |  |  |  |
| 29. | Quotations presented | | Were participant quotations presented to illustrate the themes / findings? Was each quotation identified? e*.g. participant number* | Included in section Results, page 10-17 | |
| 30. | Data and findings consistent | | Was there consistency between the data presented and the findings? | Included in section Results, page 10-17 | |
